# 3D cell subculturing pillar dish for pharmacogenetic analysis and high-throughput screening

**DOI:** 10.1016/j.mtbio.2023.100793

**Published:** 2023-09-15

**Authors:** Sang-Yun Lee, Hyun Ju Hwang, You Jin Song, Dayoung Lee, Bosung Ku, Jason K. Sa, Dong Woo Lee

**Affiliations:** a Central R & D Center, Medical & Bio Decision (MBD) Co., Ltd, Suwon, 16229, Republic of Korea; b Department of Biomedical Engineering, Gachon University, Seongnam, 13120, Republic of Korea; c Department of Biomedical Sciences, Korea University College of Medicine, Seoul, 02841, Republic of Korea

**Keywords:** 3D cell culture, Cell culture dish, Micropillar and well chips, High-throughput screening

## Abstract

A pillar dishe for subculture of 3D cultured cells on hydrogel spots (Matrigel and alginate) have been developed. Cells cultured in 3D in an extracellular matrix (ECM) can retain their intrinsic properties, but cells cultured in 2D lose their intrinsic properties as the cells stick to the bottom of the well. Previously, cells and ECM spots were dispensed on a conventional culture dish for 3D cultivation. However, as the spot shape and location depended on user handling, pillars were added to the dish to realize uniform spot shape and stable subculture, supporting 3D cell culture-based high-throughput screening (HTS). Matrigel and alginate were used as ECMs during 6-passage subculture. The growth rate of lung cancer cell (A549) was higher on Matrigel than on alginate. Cancer cell was subcultured in three dimensions in the proposed pillar dish and used for drug screening and differential gene expression analysis. Interestingly, stemness markers, which are unique characteristics of lung cancer cells inducing drug resistance, were upregulated in 3D-subcultured cells compared with those in 2D-subcultured cells. Additionally, the PI3K/Akt/mTOR, VEGFR1/2, and Wnt pathways, which are promising therapeutic targets for lung cancer, were activated, showing high drug sensitivity under 3D-HTS using the 3D-subcultured cells.

## Graphical abstract

**Figure:**
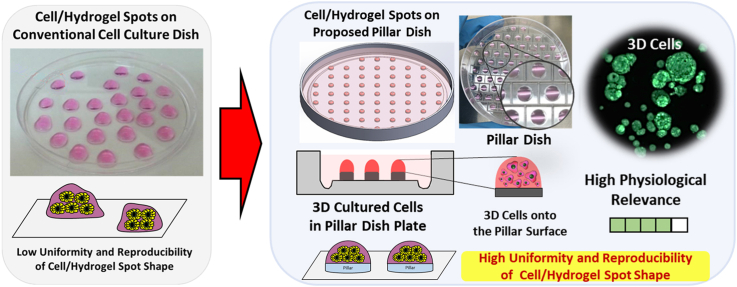

## 1 Introduction

Over the past few decades, drug development has been plagued by high failure rates [1,2]. This low success rate of drug development can be attributed to the use of *in vitro* drug screening models and inadequate preclinical test models, which do not reflect the physiological relevance and tumor microenvironment (TME) of actual patients with cancer [[3], [4], [5]]. Conventional 2D cancer cell culture systems have limitations in that even though 2D plates are coated with an extracellular matrix (ECM), such as collagen, laminin, and fibronectin, they cannot mimic the physiological patterns, such as cell–cell interactions, hypoxia, drug penetration, and resistance, noted in the tumor tissues of patient with cancer [[6], [7], [8]]. Therefore, interest in 3D cell culture models, which can better mimic the physiological relevance *in vivo*, as alternatives to the conventional 2D cell culture models has increased in recent years. The 3D cell culture method offers the advantage of simulating the physiological activity of cancer cells similar to that *in vivo* [9]. The biological and physical aspects of these 3D cell cultures activate signal transduction from the exterior to the interior of the cell and induce the expression of cell surface receptors [10]. However, changes in cancer cell signaling cascades or interactions with other cell types under 3D culture conditions remain poorly understood. In this content, many studies are underway on changes in the apoptotic cascades and signaling pathways of cancer cells in response to targeted drug treatment under 2D and 3D cell culture conditions [11,12].

In the present study, we developed a pillar dish for simple and reproducible 3D subculture of cancer cells from the initial cell culture stage (Fig. 1). Conventionally, drug efficacy assays are performed by culturing the cells under 3D conditions only at the drug screening step using cells initially subcultured under 2D conditions [[13], [14], [15]]. This approach cannot be defined as truly 3D cell culture-based drug screening, because cancer cells lose their unique *in vivo* characteristics from the early 2D subculture stage and the expression of tumor-related proteins targeted by the anticancer drugs is altered. To verify this concept, we used a lung cancer cell line derived from a single cell stock to identify changes in the expression of lung cancer-related genomic markers in cells subcultured using the proposed 3D pillar culture dish and those subcultured using the conventional 2D cell culture dish. High-throughput screening (HTS) was performed using the micropillar/microwell chip platform previously developed by our research group [[16], [17], [18], [19]]. Cells mixed with various hydrogels, such as alginate and Matrigel, were dispensed on the surface of the pillar chip and cultured uniformly under 3D conditions. Drug reactivity differed between the 2D-HTS and 3D-HTS conditions, and the reason was evaluated by cross-validating the drug response results with genomic analysis results. Genomic analysis confirmed that the generally high resistance to 70 types of anticancer drugs under 3D-HTS conditions could be attributed to the overexpression of cancer stem cell (CSC) markers related to drug resistance [20]. In addition, the higher sensitivity to drugs targeting PI3K/mTOR (XL 147, everolimus), VEGFR (regorafenib), and PORCN (LGK-974) under 3D-HTS conditions was cross-validated with genomic analysis results [21]. Overall, the proposed pillar dish subculturing 3D cultured cells is a suitable platform to supply the stable 3D cultured cells for 3D cell-based drug efficacy assays.

**Fig. 1:**
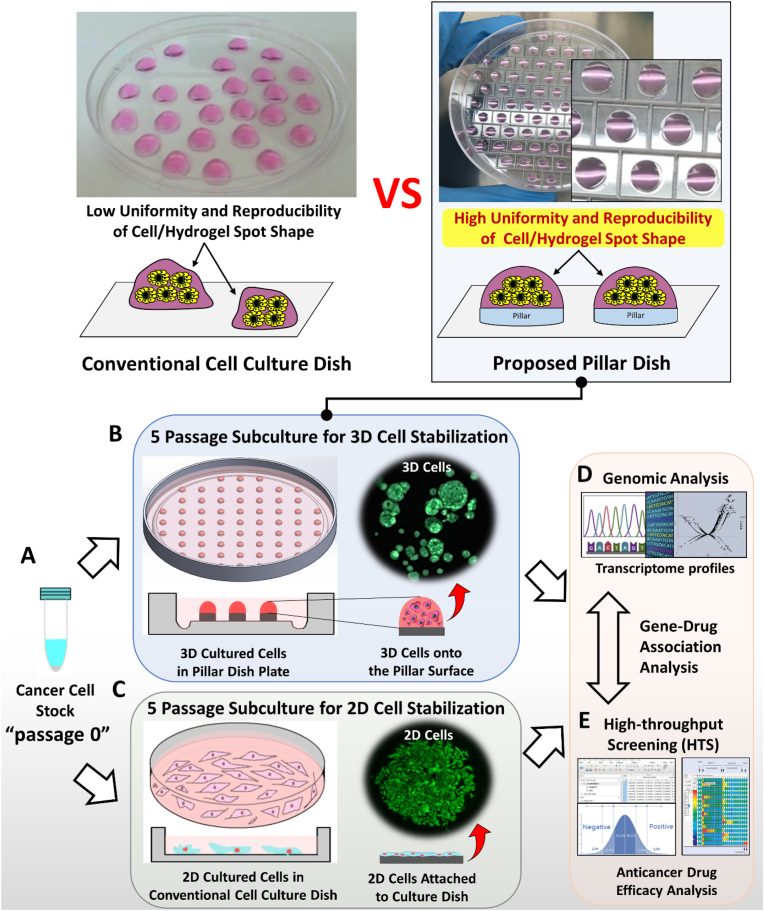
Schematic diagram of pharmacogenomic comparative analysis according to lung cancer cell culture model. A) Evenly dispensing and subculturing the cell/hydrogel mixtures are difficult on conventional cell culture dishes. B) The cell/hydrogel mixtures can be evenly dispensed, achieving high reproducibility of 3D cell subculture, on the proposed pillar dish. C) Single lung cancer cell line stock and subculture preparation. D) 3D-cultured lung cancer cells formed spheroids in the Matrigel dome, with high physiological relevance. E) 2D-cultured lung cancer cells were attached to the bottom of the dish, with low physiological relevance. F) Genomic analysis of lung cancer cells cultured under individual culture conditions. G) Anticancer drug sensitivity HTS analysis of lung cancer cells under individual culture conditions. Further, gene–drug association analysis was performed by comparing the results of genomic analysis and HTS anticancer drug sensitivity analysis.

## 2 Materials and methods

### 2.1 Preparation of the pillar dish

Using conventional cell culture dishes, it is difficult to uniformly dispense and culture cell/hydrogel mixtures under 3D conditions. In addition, the conventional method has disadvantages in that 3D cells are easily damaged during numerous steps, such as cell culture medium addition, replacement, cell recovery, and washing, and reproducible subculture is difficult. Therefore, to overcome these drawbacks, pillar dishes manufactured through plastic injection molding offer a robust and flexible platform for mammalian cell culture (Fig. 1B). Polystyrene dishes with 73 pillars (pillar diameter = 5 mm and pillar-to-pillar distance = 4 mm) were manufactured. To prevent contamination during cell culture and Matrigel polymerization, the surface of the pillar dishes was plasma-treated (80 W power, 5 × 10−4 Torr using air) for 10 s and coated with diluted laminin solution (L2020; 1 mg; Sigma, St. Louis, MO, USA) in phosphate-buffered saline (PBS). To prepare the laminin coating solution, 10 mL of PBS was mixed with a 1/100 dilution of pure laminin solution (1 mg mL^−1^). The pillar dish was similar in size to a conventional cell culture dish (70 mm × 15 mm). Plastic molding was performed with an injection molder (Sodic Plustech Inc., U.S.A.). Pillar dish plates are disposable consumables that are not reused to prevent cross-contamination during subculture. A schematic drawing and image of the fabricated pillar dish are shown in Fig. S1.

### 2.2 Gene–drug association analysis

All A549 cell lines were purchased as frozen stocks from the Korean Cell Line Bank (Seoul, South Korea) and maintained in cultures as recommended. The lung cancer cell line in the frozen stock state was rapidly thawed in a 37 °C water bath according to the recommended method, and then the prepared early-stage single cells were labeled as passage 0 (Fig. 1A). These cells were cultured until passage 6 in Roswell Park Memorial Institute (RPMI) medium 1640 (CellGro, New York, NY, USA) supplemented with 100 μg mL^−1^ streptomycin, 100 units·mL^−1^ penicillin, 250 ng mL^−1^ amphotericin B, and 10% fetal bovine serum (FBS; CellGro, Mexico City). A 2D lung cancer cell line subculture was performed by dispensing 5 × 10^5^ cells per conventional cell culture dish. Also, lung cancer cell line was separated into single cells by enzymatic treatment and prepared to contain approximately 1 × 10^4^ cells per 30 μL of 50% Matrigel (50 v/v). Then, 30 μL of the cell-Matrigel mixture was dispensed on the surface of the pillar dish designed for 3D cell culture, and gelation was performed in a 37 °C and 5% CO_2_-humidified incubator for 20 min. After fixing the 3D cells on the pillar surface, the culture medium was added, and the cells were subcultured every 4 days (Fig. 1B). For 2D culture, cells were dispensed into a conventional culture dish, maintained at 37 °C in a 5% CO_2_-humidified atmosphere, and subcultured every 4 days at 70% confluence (Fig. 1C). To solve the problem of dispensing uniformity and subculture reproducibility of cell-hydrogel mixtures in a 2D cell culture dish, we proposed a 3D subculture method in a pillar dish (Fig. 1B). After recovering the cultured A549 lung cancer cells from each culture condition, gene–drug association analysis was performed through comprehensive genomic analysis to confirm oncogenic transcript expression (Fig. 1D) and HTS analysis to measure sensitivity to the 70 anticancer drugs (Fig. 1E). In summary, a pillar dish plate was developed to optimize the subculture method according to ECMs. Also, by applying the proposed subculture method, we analyzed differences in drug response and transcriptional genomic variation of lung cancer cells according to subculture conditions (Fig. 1).

### 2.3 High-throughput drug screening

The differences in drug sensitivity between 3D-HTS and 2D-HTS conditions were compared and analyzed using lung cancer cell lines. For the 3D-HTS analysis, lung cancer cells were prepared by culturing them in 3D using a pillar dish from the initial subculture stage (Fig. S2A). 3D-subcultured lung cancer cells separated into single cells through enzymatic treatment using recovery solution I were prepared at a concentration containing approximately 100 cells per 50 nL of 50% Matrigel (50 v/v) (Fig. S2B and Table S1). The prepared cancer cell–Matrigel mixture was dispensed automatically onto a 532 micropillar chip using ASFA™ Spotter DN (Medical & Bio Decision, South Korea). The manufactured ASFA™ Spotter DN comprises an electric regulator, a syringe pump, a dispensing head, and disposable nozzles and quantitatively dispenses a liquid sample by controlling the pressure generated from the compressed air source using an electric regulator. ASFA™ Spotter DN was used to dispense 50 nL droplets of the cancer cell–Matrigel mixture on the surface of the 532 micropillar chip (Fig. S2C). To attach the Cell/Matrigel mixture to the surface of the 532 micropillar chip, the Matrigel gelation process was performed for 15 min in a 5% CO_2_ incubator maintained at 37 °C [18,22]. Then, the 532 micropillar chip containing the cells in Matrigel was sandwiched (or “stamped”) with a 532 microwell chip for 3D cell culture and drug exposure for 7 days (Fig. S2D). For live-cell staining, the staining solution was prepared by adding 1 μL of Calcein AM (C1430, Invitrogen, U.S.A.) into 7 mL of RPMI medium. The cells were incubated with the staining solution for 1 h at 37 °C in a 5% CO_2_-humidified atmosphere. Then, live-cell images with green fluorescence intensity (excitation/emission, 494/517 nm to lasers) were scanned using an optical scanner (ASFA Scanner HE, Medical and Bio Decision, Korea), as shown in Fig. S2E. The scanned images (Fig. S2F) were evaluated using an image analysis software (ASFA Ez SW, Medical and Bio Decision, Korea). Live cells showed green fluorescence. The relative cell viability following treatment with individual drugs was normalized and calculated based on the live cell area of DMSO control spots without cytotoxicity (Fig. S2G). The relative cell viability was calculated as follows:

(I) $$Relative\;cell\;viability\;\left[\%\right]=\frac{{Average\;area}_{drug}}{{Average\;area}_{DMSO\;control}}\times 100\%$$

In addition, drug reactivity was analyzed by converting the relative cell viability value into a Z-score (Table 2). Using the mean and standard deviation of the relative cell viability under treatment with the 70 drugs, the Z-score for each drug was calculated as follows:

(II) $${Z-\text{score}}_{drug}=\frac{{Relative\;cell\;viability}_{drug}-mean}{SD}$$

**Table 1:** Summary of 3D subcultured lung cancer cell proliferation rate according to ECMs.

3D Lung Cancer Cell Proliferation Rate [%]									
Subculture	1	2	3	4	5	6	Average	STDEV	CV
Matrigel	634.6	645.8	646.8	640.9	650.1	657.2	645.9	5.4	0.8
Alginate	465.1	455.6	477.2	456.7	476.3	451.7	463.7	10.9	2.4

**Table 2:** Summary of sensitive drugs under 3D-HTS conditions (Z-Score of 3D-HTS is < 1 and the linear distance is > 1).

No	Drug	Target	Relative Cell viability [%]	3D-HTS	Z-Score	3D-HTS
			2D-HTS		2D-HTS	
1	DMSO	Vehicle control	100.00	100.00	2.41	−0.13
2	AEE788 (NVP-AEE788)	EGFR	2.43	100.89	−0.82	−0.09
3	Afatinib (BIBW2992)	EGFR	1.15	102.56	−0.87	−0.02
4	BMS-599626 (AC480)	EGFR	43.20	127.70	0.53	1.06
5	Erlotinib HCl	EGFR, HER2	45.53	117.10	0.60	0.61
6	Dacomitinib (PF299804,PF-00299804)	EGFR	4.09	109.62	−0.77	0.29
7	Gefitinib (Iressa)	EGFR	9.50	83.83	−0.59	−0.82
8	Lapatinib	HER1/EGFR	0.26	53.94	−0.90	−2.11
9	Neratinib (HKI-272)	EGFR	24.35	131.12	−0.10	1.21
10	CI-1033 (Canertinib)	EGFR, HER2	29.54	113.00	0.07	0.43
11	CO-1686	EGFR, HER2	1.12	111.65	−0.87	0.37
12	BKM120 (NVP-BKM120)	mTOR	4.31	119.83	−0.76	0.73
13	BYL719	AKT1/2/3	6.16	71.72	−0.70	−1.35
14	XL147	PI3K/mTOR	8.89	41.65	−0.61	−2.64
15	Everolimus (RAD001)	PI3K	48.00	62.52	0.69	−1.74
16	AZD2014	PI3K	14.85	124.21	−0.41	0.91
17	PF-05212384 (PKI-587)	mTOR	12.73	109.42	−0.48	0.28
18	XL765 (SAR245409)	PI3K/mTOR	14.84	106.63	−0.41	0.16
19	BEZ235	PI3K	30.38	75.99	0.10	−1.16
20	AZD5363	PI3K/mTOR	5.66	96.35	−0.72	−0.29
21	ABT-199 (GDC-0199)	Bcl-2	4.16	106.37	−0.77	0.15
22	ABT-888 (Veliparib)	PARP	94.62	101.98	2.23	−0.04
23	AUY922 (NVP-AUY922)	HSP (e.g. HSP90)	11.90	117.39	−0.51	0.62
24	Axitinib	VEGFR1/2/3, PDGFRβ and c-Kit	62.44	93.55	1.16	−0.41
25	AZD4547	FGFR1/2/3	6.04	68.38	−0.70	−1.49
26	AZD6244 (Selumetinib)	MEK1	13.94	98.79	−0.44	−0.18
27	LGK-974	PORCN	45.28	65.18	0.60	−1.63
28	BGJ398 (NVP-BGJ398)	FGFR1/2/3	40.05	108.39	0.42	0.23
29	Bortezomib (Velcade)	Proteasome	0.78	99.46	−0.88	−0.15
30	Cediranib (AZD2171)	VEGFR, FIT	41.62	93.29	0.47	−0.42
31	Crizotinib (PF-02341066)	Met, ALK	0.21	83.71	−0.90	−0.83
32	Dasatinib (BMS-354825)	Bcr-Abl	0.29	97.98	−0.89	−0.22
33	Dovitinib (TKI-258)	Flt3, c-Kit, FGFR1/3, VEGFR1/2/3, PDGFRα/β	2.32	109.48	−0.83	0.28
34	Imatinib (Gleevec)	v-Abl,/2/bl, c-Kit and PDGFR	17.25	101.08	−0.33	−0.08
35	INCB28060	Met	99.82	105.92	2.40	0.13
36	LY2835219	CDK4/6	0.00	104.67	−0.90	0.07
37	DMSO	Vehicle control	90.87	110.86	2.11	0.34
38	Cabozantinib (XL184)	VEGFR2, c-Met, Ret, Kit, Flt-1/3/4, Tie2, AXL	33.54	117.28	0.21	0.62
39	Foretinib (XL880)	HGFR, VEGFR, mostly for Met and KDR	0.19	67.17	−0.90	−1.54
40	Ibrutinib	Btk, modestly potent to Bmx, CSK, FGR, BRK, HCK, less potent to EGFR, Yes, ErbB2, JAK3	1.35	118.02	−0.86	0.65
41	vemurafenib	B-RafV600E	20.06	125.60	−0.24	0.97
42	trametinib	MEK1/2	21.47	102.57	−0.19	−0.02
43	LDE225 (NVP-LDE225, Erismodegib)	Smoothened	56.25	89.87	0.96	−0.56
44	LDK378	ALK	0.00	58.08	−0.90	−1.93
45	LEE011	CDK4/6	51.67	122.27	0.81	0.83
46	Nilotinib (AMN-107)	Bcr-Abl	56.07	118.79	0.95	0.68
47	Olaparib (AZD2281)	PARP1/2	37.94	115.78	0.35	0.55
48	Panobinostat (LBH589)	HDAC	3.88	98.51	−0.78	−0.19
49	Pazopanib HCl	VEGFR1/2/3, PDGFR. FGFR, c-Kit	15.29	82.24	−0.40	−0.89
50	PD 0332991 (Palbociclib HCl)	CDK4/6	2.50	103.25	−0.82	0.01
51	PF-04449913	HSP90	79.62	120.25	1.73	0.74
52	Sotrastaurin (AEB071)	PKC	9.61	127.05	−0.59	1.04
53	Sunitinib Malate (Sutent)	VEGFR2 and PDGFRβ	0.44	145.45	−0.89	1.83
54	Tandutinib (MLN518)	FLT3, PDGFR, FGFR, c-Kit	4.88	124.95	−0.74	0.95
55	Tivozanib (AV-951)	VEGFR, c-Kit, PDGFR	3.45	126.12	−0.79	1.00
56	Vismodegib (GDC-0449)	Hedgehog/smoothend	84.88	135.80	1.91	1.41
57	PHA-665752	c-Met inhibitor	0.70	152.10	−0.88	2.12
58	Dabrafenib	BRAFV600	7.44	104.66	−0.66	0.07
59	Regorafenib	VEGFR1/2/3, PDGFR, Kit, RET and Raf-1	0.33	24.48	−0.89	−3.38
60	Bosutinib	dual Src/Abl	1.52	120.82	−0.85	0.77
61	Carfilzomib	Proteasome	5.90	100.08	−0.71	−0.12
62	Ruxolitinib	JAK1/2	20.07	92.76	−0.24	−0.44
63	Vandetanib	VEGFR2	53.28	121.35	0.86	0.79
64	TMZ	alkylating agent	79.39	122.68	1.73	0.85
65	Amorolfine	morpholine antifungal drug, fungal enzymes D14 reductase and D7-D8 isomerase	102.84	116.49	2.50	0.58
66	Mevastatin	HMG-CoA reductase inhibitor	88.37	98.91	2.02	−0.17
67	Amiodarone	antiarrhythmic medication. β- and γ-secretase	48.89	99.38	0.72	−0.15
68	Fluvastatin Na	Anticholesterol agent. HMG-CoA inhibitor	64.15	98.83	1.22	−0.18
69	Mycophenolic acid	inosine-5′-monophosphate dehydrogenase inhibitor	24.47	132.11	−0.09	1.25
70	Raloxifene HCl	estrogen receptor inhibitor	21.44	128.87	−0.19	1.12
71	Astemizole	histamine receptor ligand	24.43	99.87	−0.09	−0.13
72	Fenretinide	retinoic acid receptor ligand	0.14	75.31	−0.90	−1.19

**Table 3:** Summary of relative cell viability and Z-Score in 2D-HTS and 3D-HTS models.

No	Drug	Target	Linear Distance
14	XL147	PI3K/mTOR	1.44
15	Everolimus	PI3K	1.72
27	LGK-974	PORCN	1.57
59	Regorafenib	VEGFR1/2/3, PDGFR, Kit, RET and Raf-1	1.76

In addition, drugs showing specific sensitivity in 3D-HTS with a Z-score value of −1 or less, linear distance of >1 based on line (1), and the distance between lines (1) and (2) = 1 were selected (Fig. 3C and Table 1). The linear distance was calculated as follows:

(III) $${\text{Linear}\;\text{Distance}}_{drug}=\frac{-{Z-score}_{3D\;HTS}+{Z-score}_{2D\;HTS}}{\sqrt{2}}$$

For the 2D-HTS analysis, the lung cancer cells were cultivated on a conventional culture dish and separated into single cells by enzymatic treatment (Fig. S2H). The cells were mixed with fresh cell culture medium without Matrigel and prepared at a concentration of 100 cells per 450 nL of medium (Fig. S2I). The cells were dispensed into a 532 microwell chips at the volume of 450 nL using ASFA™ Spotter ST (Fig. S2J), and then the 70 individual drugs were dispensed at the volume of 450 nL for drug treatment. The microwell chip, in which cells and drugs were dispensed, was placed in an incubator for 7 days to verify the drug response (Fig. S2K). For live-cell staining, the supernatant was removed from the microwell, followed by the addition of a live-cell staining solution. Images of live cells attached to the bottom of the 532 microwell chip showing green fluorescence were obtained using an optical scanner (ASFA Scanner HE, Medical and Bio Decision, Korea), as shown in Fig. S2L. Subsequently, 2D-HTS analysis based on relative cell viability calculated from the obtained live-cell image was performed in the same manner as 3D-HTS analysis. The relative cell viability and Z-score values are listed in Table 2, along with the drug name and drug-specific targets.

### 2.4 Preparation of micropillar and microwell chips

Micropillar and microwell chips manufactured via plastic injection molding are robust and flexible platforms for mammalian cell culture, enzymatic reactions, viral infection, and compound screenings. The micropillar chip composed of PS-MA contained 532 micropillars (pillar diameter = 0.75 mm and pillar-to-pillar distance = 1.5 mm) (Fig. S2C). Similarly, the microwell chip contained 532 microwells (well diameter = 1.2 mm and well-to-well distance = 1.5 mm) (Fig. S2J). Both micropillar and microwell chips were similar to conventional microscopic glass slides in terms of size (75 × 25 mm), as shown in Fig. S5. PS-MA, a widely used biocompatible plastic, was used to fabricate the micropillar and microwell chips. For Matrigel polymerization, the surface of the pillar was plasma-treated for 10 s (80 W power, 5 × 10−4 Torr using air) and coated with a diluted laminin solution (L2020-1 mg, Sigma, St. Louis, MO, USA) in PBS, as previously mentioned. Plastic molding was performed using an injection molder (Sodic Plustech Inc., U.S.A.). Micropillar and microwell chips are disposable consumables that are not reused after HTS analysis. Fig. S2 summarizes the methods for 2D and 3D-HTS based drug response analysis using micropillar and microwell chips.

### 2.5 Whole-transcriptome sequencing

Samples were collected in 1 mL of TRIzol Reagent per 1 pillar dish (5–10 × 10^5^ cells seeding) and stored at −80 °C. RNA was extracted using the RNA mini prep Kit (Qiagen). One μg RNA from total RNA samples (4–8 μg per sample) was used to library preparation using TruSeq RNA Sample Rrep Kit (Illumina). The TruSeq RNA Sample Prep Kit was used to prepare RNA-Seq libraries, and the HiSeq4000 (Illumina) platform was used to generate 100 bp paired-end reads. Whole-transcriptome sequencing reads were aligned to the reference human genome (GRCh38) using STAR, and gene expression levels were determined and quantified based on the fragments per kilobase per million (FPKM) values. The FPKM values were log_2_ transformed and subjected to further analyses.

### 2.6 Differentially expressed gene analysis

Differentially expressed gene (DEG) analysis was performed using DESeq2, and significant DEGs were determined based the significance threshold of p < 0.05 and log_2_ fold change enrichment threshold of |Log_2_ FC| > 5. To acquire pathway-level activities, single-sample gene set enrichment analysis (ssGSEA) was performed using the gene set database *c2.all.v2022.1.Hs.symbols.gmt.*

## 3 Results and discussion

### 3.1 Optimized subculture protocol according to the ECMs (Alginate and Matrigel)

During the six passages of cancer cells on a pillar dish, the subculture method was optimized to increase the yield of cancer cells and efficiently isolate 3D colony cancer cells into single cells (Fig. S3). The subculturing protocol is depending on ECM. The culture medium of cancer cells cultured in 3D with Matrigel was removed and washed three times with 7 mL of cold PBS. Then, 10 mL of cold recovery solution I was dispensed, and the pillar dish was placed in an ice bath and tilted for 1 h. Recovery solution I containing the isolated single cancer cells was collected, and 10 mL of PBS washing and centrifugation (2000 rpm, 4 °C, 3 min) were repeated twice. The harvested cell pellet was resuspended in 1 ml of culture medium and the number of single cancer cells was counted. It was then mixed with matrigel and subcultured in a pillar dish. (Fig. S3A). After removing the culture medium of cancer cells cultured in 3D with alginate, 10 mL of recovery solution II was dispensed. The pillar dish was stabilized in a CO_2_ incubator for 3 min, then placed in a bead warming bath maintained at 37 °C and tilted for 7 min. Recovery solution II containing the isolated single cancer cell was collected, and the 10 mL of PBS washing and centrifugation process (2000 rpm, 26 °C, 3 min) was repeated twice. The collected cell pellet was resuspended in 1 mL of culture medium and the number of single cancer cells was counted. It was then mixed with alginate, and subcultured on a pillar dish (Fig. S3B). It was difficult to separate the 3D cultured cancer cells into single cells if the tilting process was not included during the subculture process (Fig. S4A). In addition, since the temperature conditions for degelation are different depending on the ECMs, an ice bath maintained at 4 °C was applied for matrigel (Fig. S4C), and a bead warming bath maintained at 37 °C was applied for alginate (Fig. S4B) to each tilting process, thereby efficiently separating into single cancer cells. The detailed recipe for recovery solutions is listed in Supplemental Table 1.

### 3.2 Stable growth rate in the subculture of lung cancer cell using the pillar dish

Lung cancer cells were subcultured under different Matrigel and alginate ECM conditions using the proposed pillar dish to stabilize cells under the 3D culture conditions. Subculture was repeated at 4-day intervals for six times in total. Growth rates were calculated for all subcultures. Alginate and Matrigel growth rate coefficient of variation (CV) were less than 5%, indicating stable performance of the subculture protocol of 3D grown cells in the pillar dish. Lung cancer cells were subcultured for 5 passages to stabilize and then used for drug screening. Since cells of 6 passages were used for drug screening, we verified the proliferation rate of lung cancer cells up to 6 passages. The lung cancer cell proliferation rate was indicated as the percentage of the total number of live cells recovered after culturing for 4 days compared with the number of cells initially seeded. Through bright field (BF) image scan, the cells that were initially seeded and proliferated by forming spheroids after 3D culture for 4 days were confirmed (Fig. 2A). On the BF image, lung cancer cells, which were initially at the single-cell status, formed spheroids of a more mature size under Matrigel ECM conditions than under alginate ECM conditions after 3D culture for 4 days. To quantitatively analyze the spheroid formation and proliferation of lung cancer cells under the Matrigel ECM conditions, the 3D-cultured cells were recovered and the number of proliferated live cells was counted. Lung cancer cells cultured for 4 days under the Matrigel ECM conditions achieved the average proliferation rate of 645%, compared with 463.7% under the alginate ECM conditions (Fig. 2B). The proliferation rates of 3D subcultured lung cancer cells according to the ECMs are shown in Table 1. In HTS and genomic analyses, Matrigel was used due to high growth rate.

**Fig. 2:**
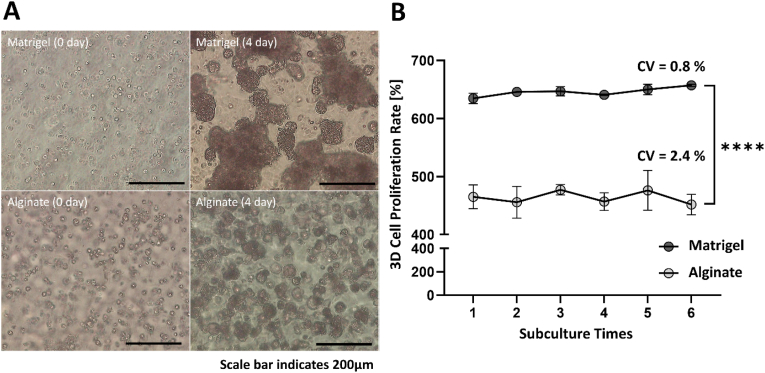
Measurement of the proliferation rate of subcultured lung cancer cells according to ECM. A) BF images of lung cancer cells cultured for 0–4 days according to ECM. B) Subculture proliferation rate of lung cancer cells according to ECM. The rate was approximately 645% on Matrigel and approximately 463% on alginate. The coefficient of variation (CV) values of 0.8% and 2.4% for the Matrigel and Alginate conditions, respectively, show high subculture performance.

**Fig. 3:**
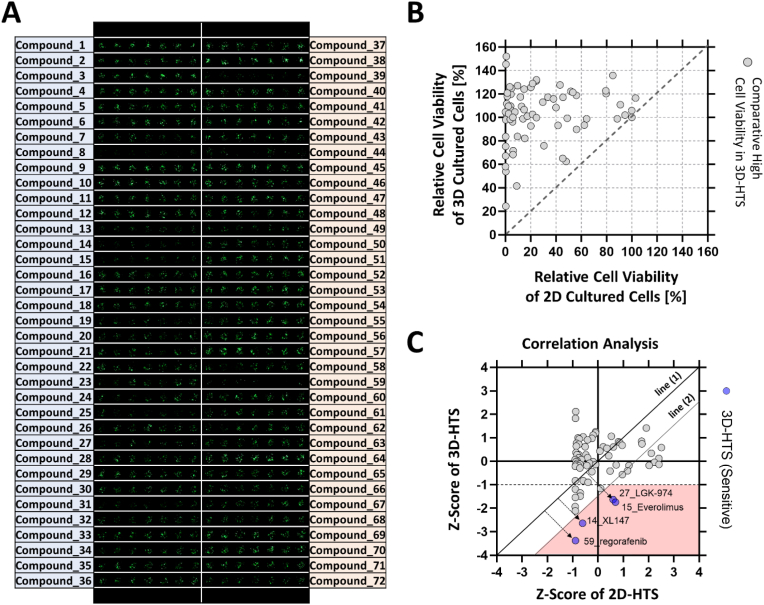
HTS anticancer drug sensitivity analysis results using micropillar/microwell chip. A) Scanning image of micropillar chip exposed to 72 compounds (including two DMSO controls). B) Comparative analysis of anticancer drug sensitivity according to cell culture model based on the relative cell viability value. C) Comparative analysis of anticancer drug sensitivity according to cell culture model based on the Z-score value.

### 3.3 Comparative analysis of drug response in 2D and 3D cultured cells

In 3D-cell based assays, culturing cells under 3D conditions not only maintains the unique characteristics of cancer cells but also promotes growth and improves drug resistance [23,24]. To confirm this, the response to 70 drugs was quantitatively analyzed using lung cancer cell lines grown under different 2D and 3D culture conditions. Cancer drugs administered in the current clinical trials and standard target oncology drugs were selected. From the drug library, 70 anticancer drugs with well-known targets, including epidermal growth factor receptors (EGFR), phosphoinositide 3-kinase (PI3K), mechanistic target of rapamycin (mTOR), vascular endothelial growth factor receptors (VEGFR), *MET* gene, tyrosine-protein kinase Met (c-Met), and fibroblast growth factor receptors (FGFR), were selected. According to public data provided by the US Food and Drug Administration (FDA), these drugs were in phase III or IV trials or were approved oncology drugs. In addition, anticancer drugs with various pharmacological mechanisms were used to analyze the differences in transcriptional genomic variations and drug response of lung cancer cells according to subculture conditions. The 70 selected drugs (including DMSO controls) were dispensed onto a 532 microwell chip. On the 532 microwell chip layout, the 70 individual drugs were dispensed with six technical replicates, and the responses of live cells to different drugs were scanned (Fig. 3A). The 532 microwell chip was divided into 72 regions, and individual drugs at a single concentration of 20 μM were dispensed into each region. Compounds 1 and 37 refer to the DMSO control group; as control, DMSO diluted in the growth medium (final concentration of 0.5% v/v) was used for all 70 drugs. Drug reactivity was compared based on relative cell viability between 2D-HTS and 3D-HTS conditions. The relative cell activity values of 70 anticancer drugs were distributed in the area with a slope of 1 or more. A slope value of 1 or higher indicates that the relative cell viability rate is distributed more on the y-axis, representing 3D-HTS. Therefore, based on the comparison of relative cell viability, comparatively higher cell activity values were measured under 3D-HTS than under 2D-HTS conditions for all 70 drugs (Fig. 3B). Since the relative cell viability was generally higher under 3D-HTS conditions, further comparative analysis was performed with Z-score, which is the relative drug efficacy value, under different drug screening conditions (Fig. 3C). The Z-score representing relative drug efficacy was calculated using the relative cell viability value. Drugs were judged as exhibiting a particularly sensitive response under the 3D-HTS condition when their Z-score was below −1 and when the linear distance exceeded 1 from line 1 simultaneously (Table 1). In Z-Score analysis, four drugs targeting VEGFR1/2/3, PDGFR, Kit, RET, and Raf-1 (regorafenib); PI3K/mTOR (XL 147, everolimus); and PORCN (LGK-974) showed a more sensitive response under 3D-HTS conditions (blue dots).

### 3.4 Global transcriptional expression shift analysis according to the cell culture model

As described earlier, different drug responses were observed depending on the cell culture model; therefore, to identify the potential underlying transcriptomic signatures regulating the distinct pharmacological responses of lung cancer cells in the 2D and 3D models, we performed DEG analysis. The cut-off for significance was determined based on a P-value of <0.05 and absolute log2 fold change of >5. (Fig. 4A and B). Using the cell extraction protocols described in Materials and methods section, target cells could be successfully collected from both conventional 2D cell culture model and the proposed 3D cell subculture model using pillar dishes and used for RNA sequencing. Essential molecules related to the rat sarcoma (RAS) and retinoblastoma protein (RB1) pathways were upregulated and hepatocyte growth factor (HGF) was overexpressed in a lung cancer cells subcultured under 3D conditions. As a result of upregulation of these signaling pathways, cascades related to angiogenesis, cell differentiation, and ECM were activated in 3D-subcultured lung cancer cells (Fig. 4B and C). In contrast, under 3D cell subculture conditions, the activity of oxygen transport and myelocytomatosis (Myc)-based signaling pathways related to hypoxia was inhibited [25,26]. Therefore, the TME, such as the activation of signaling pathways related to tumorigenesis and hypoxia, was differently stimulated under the conventional 2D and proposed 3D cell subculture conditions.

**Fig. 4:**
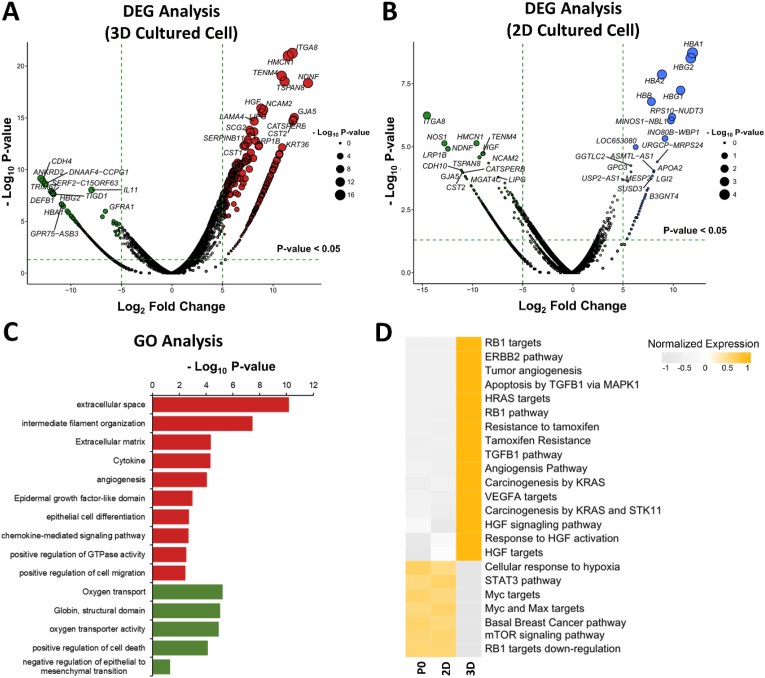
Transcriptome profiling according to lung cancer cell culture model. A, B) Volcano plot of differentially expressed genes showing the magnitude and significance of gene upregulation in 3D- and 2D-cultured lung cancer cells. C) GO analysis of hallmark genes enriched in 3D-cultured lung cancer cells. D) Analysis of hallmark genes enriched in 3D cultured lung cancer cells through differential gene expression analysis between lung cancer cell stocks and subculture models.

### 3.5 Genome expression and drug response association analysis

The results of earlier drug screening and genome expression analyses were combined to validate the differences in drug responses according to cell culture conditions. In particular, many studies have previously reported that genomic expression differences were observed in 2D and 3D cell culture conditions [27,28]. Practically, early passage 3D cultured cells show different genomic expressions depending on the 3D culture times and passages [[29], [30], [31], [32]]. As such, genomic expression changes are progressing in the early 3D subculture stage cells according to subculture time and passage. In this study, we focused to analyzed the genomic expression and drug screening results at the endpoint where the genome expression change of cells was stabilized through a sufficient 3D subculture process. The cell genomic expression differences and drug response according to various subculture conditions (subculture time and passage) is being planned as a follow-up study. Typically, signaling pathways related to the stemness of cancer cells are activated under 3D cell subculture conditions, which induce drug resistance [33,34]. The activity of signaling pathway related to the stemness of cancer cells was significantly increased in 3D-subcultured lung cancer cells using the proposed pillar dish compared to that in 2D-cultured cells using the conventional method (p = 6.03 × 10^−4^) (Fig. 5A). Specifically, the transcript expression levels of stemness markers, such as CD44, ATP-binding cassette subfamily G member 2 (ABCG2), THY1 (CD90), and ALCAM (CD166), which are promising therapeutic targets for lung cancer and are known to induce drug resistance, were increased [[35], [36], [37]] (Fig. 5B). In addition, the transcript expression of lung CSC-related markers, such as sex-determining region Y-box 2 (SOX2) and octamer-binding transcription factor 4 (OCT4), which are known to increase along with increased expression of stemness markers, were increased in 3D-subcultured cells [38,39]. These genomic analysis results were consistent with drug screening results, which revealed generally higher drug resistance under 3D-HTS conditions in drug efficacy assays.

**Fig. 5:**
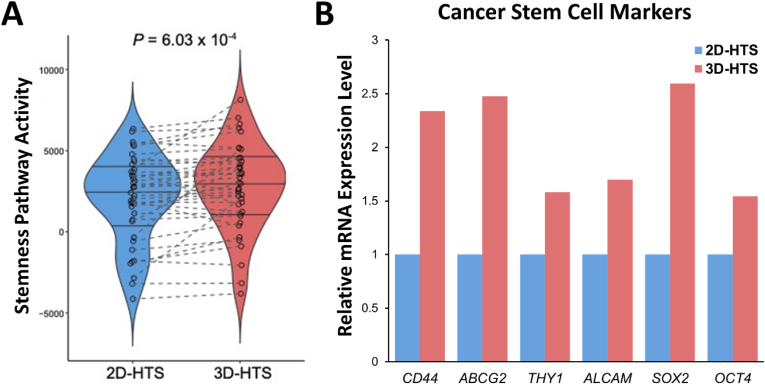
Gene expression analysis of stemness markers related to increased drug resistance in 3D-cultured lung cancer cells. A) Violin plots indicating the activity level of stemness-related pathways using ssGSEA. B) Differences in transcript expression between 2D- and 3D-cultured lung cancer cells.

When drug efficacy was analyzed based on the Z-score and linear distance, four drugs with more sensitive efficacy were identified under 3D-HTS conditions (Fig. 6A). In particular, drugs targeting PI3K/mTOR (XL 147, everolimus), VEGFR (regorafenib), and PORCN (LGK-974) were selected as those showing a more sensitive response under 3D-HTS conditions; therefore, these responses were comprehensively analyzed in terms of the modulation of the target pathway activity based on genome analysis. The PI3K/mTOR [40,41], VEGFR [42,43], and Wnt [44,45] pathways, which are promising therapeutic targets in lung cancer, were significantly activated under 3D-HTS conditions (Fig. 6B and C). In other words, the four types of drugs targeting the corresponding pathways showed a more sensitive response under the 3D-HTS condition. Therefore, for 3D-HTS drug efficacy assays, culturing and preparing cells under 3D conditions is crucial such that the unique characteristics of cancer cells are well preserved from the initial cancer cell culture stage. In addition, drug efficacy analysis using Z-score and linear distance showed that drugs with a specifically sensitive response under 3D-HTS conditions were successfully cross-validated using genomic results. Among the drugs predicted to be sensitive to 3D subcultured lung cancer cells, phase 1 clinical trials of XL147 [46] and phase 2 clinical trials of Everolimus [47] were conducted in lung cancer patients. In the case of LGK-974, drug efficacy was verified in lung cancer organoids and *in vivo* models [48]. Therefore, we plan to conduct a study to analyze the efficacy of the selected 4 drugs in lung cancer patient-derived organoids and *in vivo* models. In addition, since not only cell proliferation rate but also drug response and transcriptional genomic variations will be different depending on ECMs, further studies will be conducted to verify the difference in cell characteristics according to ECMs.

**Fig. 6:**
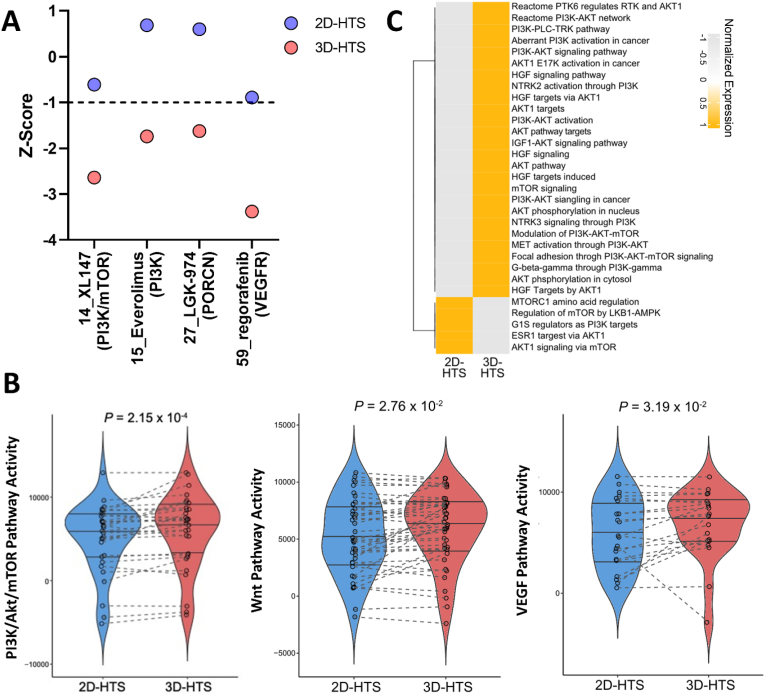
Sensitive drug selection under 3D-HTS conditions and cross-validation through genomic analysis. A) Selection of drugs showing sensitive response under 3D-HTS conditions. B) Analysis of hallmark genes enriched in 3D-cultured lung cancer cells through differential gene expression analysis between 2D-HTS and 3D-HTS models. C) Violin plots indicating the activity levels of the PI3K/Akt/mTOR, Wnt, and VEGF pathways using ssGSEA.

## 4 Conclusions

To maintain the unique physiological characteristics of lung cancer cells, a pillar dish was developed, which can be used to subculture cancer cells under 3D conditions from the initial subculture stage. Lung cancer cell lines were successfully subcultured in more than 5 passages under the conventional 2D and the proposed 3D subculture conditions. The subcultured cells of more than 5 passages were used for drug screening and genomic analysis. Responses to 70 anticancer drugs were quantified based on relative cell viability, which was converted into a Z-score, reflecting relative drug efficacy according to individual screening conditions. An overall higher drug resistance was confirmed in 3D-HTS, and four drugs (XL 147, everolimus, regorafenib, and LGK-974) showing specifically higher sensitivity under these conditions based the Z-score and linear distance were selected. In addition, differences were noted in gene expression depending on the cell culture model, consistent with the results of drug screening analysis. As such, lung CSC-related pathways that induce drug resistance were overexpressed in 3D-subcultured cells, and specific target pathways that are promising therapeutic targets for lung cancer were activated. In other words, both genome analysis and drug efficacy screening revealed marked differences between cells subcultured using the proposed 3D pillar dish and the conventional 2D cultivation method. Therefore, the proposed 3D-HTS drug efficacy analysis method based on 3D lung cancer cell culture in the present study may be a more appropriate model for application in lung cancer-related drug discovery and development research. In the future, this model can be applied in comprehensive studies aimed at new drug discovery using lung cancer-derived cells and analysis of drug efficacy based on clinical treatment outcomes.

## CRediT (contributor roles taxonomy) author statement

Sang-Yun Lee: scientific conceptualization, experimental design, bench work, collected and analyzed data, manuscript writing. Hyun Ju Hwang: collected and analyzed data, manuscript writing. You Jin Song: collected and analyzed data. Dayoung Lee: collected and analyzed data. Bosung Ku: support experiment and reviewed the manuscript. Jason K. Sa: co-supervised the work and reviewed the manuscript. Dong Woo Lee: scientific conceptualization, designed and supervised the work, and reviewed the manuscript.

## Funding

This research was supported by the Commercialization Promotion Agency for R&D Outcomes(COMPA) funded by the Ministry of Science and ICT(MSIT) (No. 1711196063, Development of high-speed, high-capacity cancer organoid imaging-based anticancer drug/radiation susceptibility screening technology) . This work was supported by the 10.13039/501100003725National Research Foundation of Korea (NRF) grant funded by the Korean government (MSIT) (No. 2020R1I1A306655012).

## Declaration of competing interest

The authors declare that they have no known competing financial interests or personal relationships that could have appeared to influence the work reported in this paper.

## Figures and Tables

**figs1:**
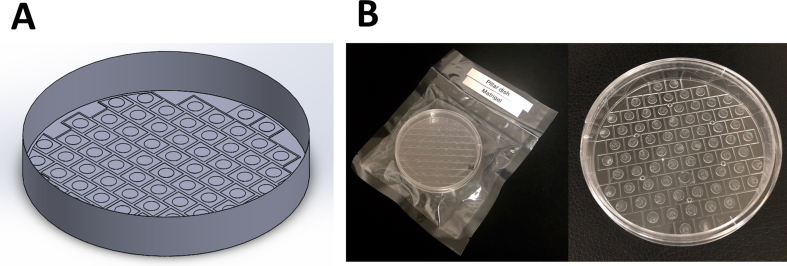


**figs2:**
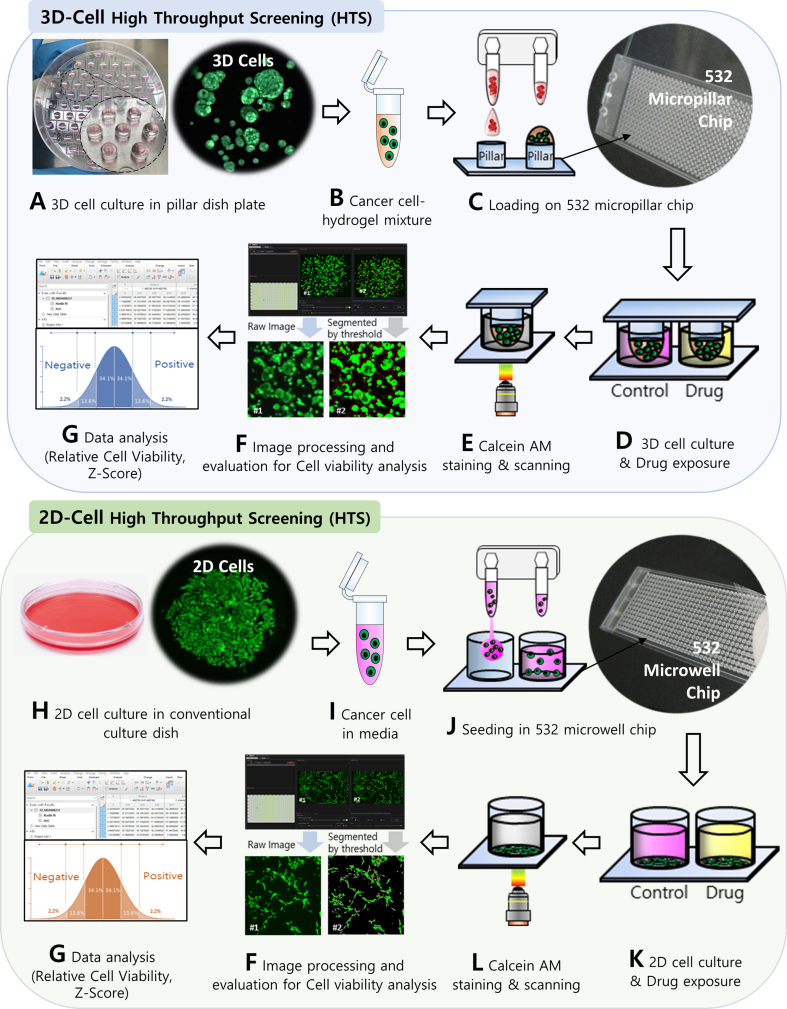


**figs3:**
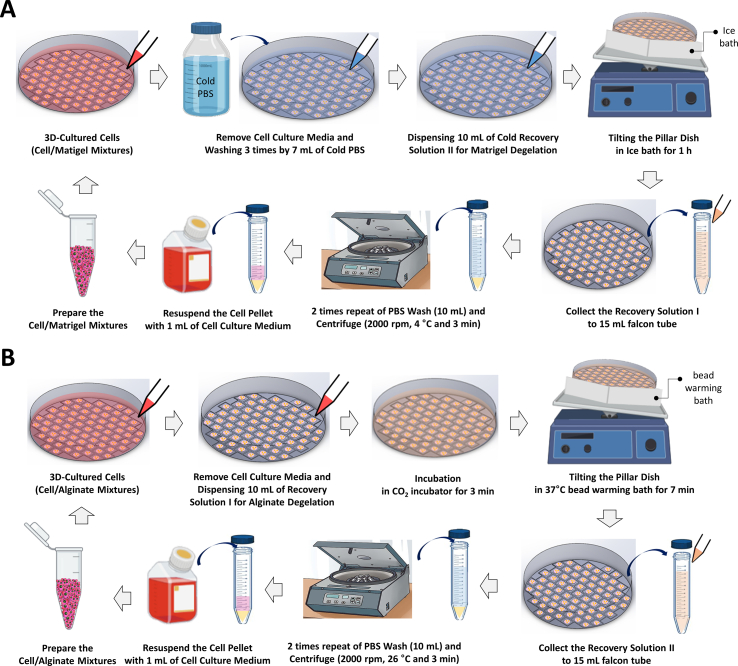


**figs4:**
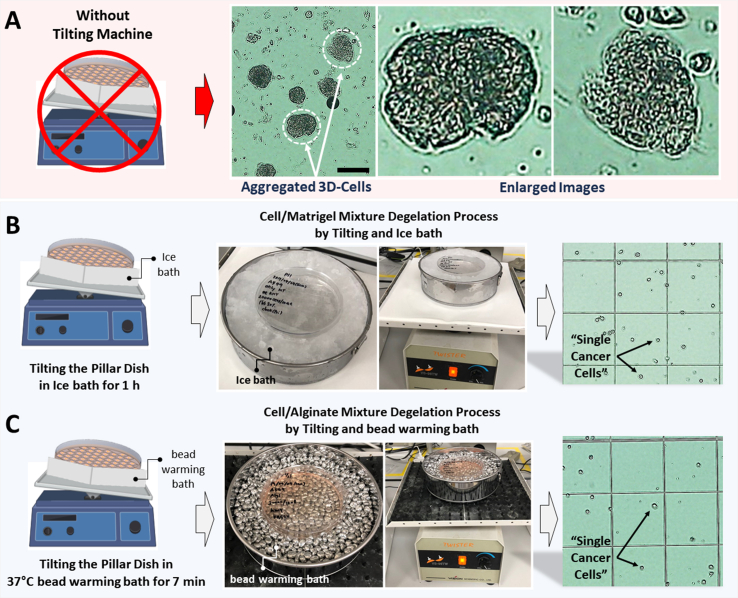


**figs5:**
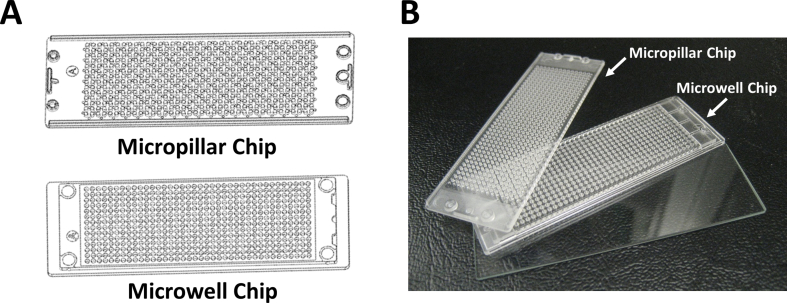


**figs6:**
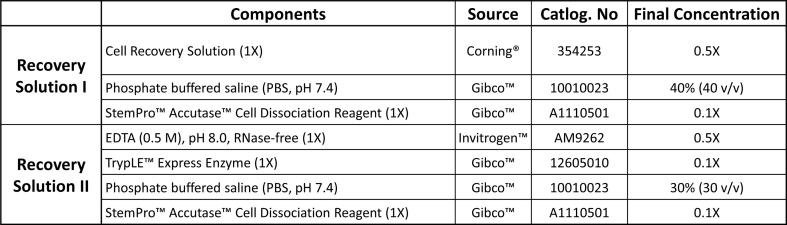


## Data Availability

Data will be made available on request.
